# Association between Landscape Factors and Spatial Patterns of *Plasmodium knowlesi* Infections in Sabah, Malaysia

**DOI:** 10.3201/eid2202.150656

**Published:** 2016-02

**Authors:** Kimberly M. Fornace, Tommy Rowel Abidin, Neal Alexander, Paddy Brock, Matthew J. Grigg, Amanda Murphy, Timothy William, Jayaram Menon, Chris J. Drakeley, Jonathan Cox

**Affiliations:** London School of Hygiene and Tropical Medicine, London, UK (K.M. Fornace, N. Alexander, C.J. Drakeley, J. Cox);; Infectious Diseases Society, Kota Kinabalu, Malaysia (T.R. Abidin, M.J. Grigg, T. William);; Menzies School of Health Research, Kota Kinabalu (T.R. Abidin, M.J. Grigg, T. William);; University of Glasgow, Glasgow, UK (P. Brock);; Menzies School of Health Research, Darwin, Northern Territory, Australia (M.J. Grigg, A. Murphy);; Jesselton Medical Centre, Kota Kinabalu (T. William);; Sabah Department of Health, Kota Kinabalu (T. William, J. Menon)

**Keywords:** *Plasmodium knowlesi*, deforestation, malaria, parasites, zoonoses, mosquitoes, macaques, Borneo, Malaysia

## Abstract

The zoonotic malaria species *Plasmodium knowlesi* has become the main cause of human malaria in Malaysian Borneo. Deforestation and associated environmental and population changes have been hypothesized as main drivers of this apparent emergence. We gathered village-level data for *P. knowlesi* incidence for the districts of Kudat and Kota Marudu in Sabah state, Malaysia, for 2008–2012. We adjusted malaria records from routine reporting systems to reflect the diagnostic uncertainty of microscopy for *P. knowlesi*. We also developed negative binomial spatial autoregressive models to assess potential associations between *P. knowlesi* incidence and environmental variables derived from satellite-based remote-sensing data. Marked spatial heterogeneity in *P. knowlesi* incidence was observed, and village-level numbers of *P. knowlesi* cases were positively associated with forest cover and historical forest loss in surrounding areas. These results suggest the likelihood that deforestation and associated environmental changes are key drivers in *P. knowlesi* transmission in these areas.

## Abstract 2

Forest loss and other environmental changes correlate with increased malaria incidence.

Since the initial description of a large cluster of human infections with the zoonotic malaria *Plasmodium knowlesi* in Malaysian Borneo in 2004, increasing numbers of *P. knowlesi* cases have been identified throughout Southeast Asia (*1**,**2*). Although most persons infected with *P. knowlesi* respond to treatment, infection can cause severe and fatal disease (*3*). Understanding the distribution of *P. knowlesi* malaria and risk factors associated with this disease is critical for designing appropriate public health interventions.

Carried by long- and pig-tailed macaques (*Macaca fascicularis* and *M. nemestrina*), the *P. knowlesi* parasite has a geographic range limited by the distribution of mosquito vectors and simian hosts (*2*). Within this range, risk for *P. knowlesi* infection in humans is highly variable. Although sporadic *P. knowlesi* cases have been reported in several Southeast Asia countries, *P. knowlesi* is the most common cause of human malaria Malaysian Borneo, the portion of the country that lies on the island of Borneo (*1**,**2*). In the state of Sabah, suspected *P. knowlesi* notifications increased from 2% (59/2,741) of total malaria notifications in 2004 to 62% (996/1,606) in 2013 (*4**,**5*).

Molecular studies indicate that zoonotic *P. knowlesi* is not a newly emergent malaria species and is likely to predate human settlement in Southeast Asia (*6*). *P. knowlesi* was first described in macaques in the 1930s, and the first naturally acquired human case was reported in 1965 in peninsular Malaysia (*7**,**8*). However, true incidence and effects of *P. knowlesi* are poorly understood because of its frequent misidentification by microscopy as other human malaria species and because of limited availability of *P. knowlesi–*specific molecular diagnostic capabilities. *P. knowlesi* appears microscopically similar to the human malaria species *P. malariae* but can also be misdiagnosed as *P. falciparum* or *P. vivax* (*3*). The extent to which improved detection has contributed to recent increases in numbers of human cases is difficult to determine; however, the rise in *P. knowlesi* relative to other malaria species strongly suggests that *P. knowlesi* transmission has increased in Malaysian Borneo (*4**,**5*).

Land use changes, such as deforestation and agricultural expansion, have been proposed as the main drivers of this apparent emergence (*3*). Deforestation and related forest activities have been associated with changes in malaria vector populations and related disease incidence globally (*9*). Changes in vegetation, microclimate, and soil composition can affect the species composition and abundance of mosquito populations (*10*). In Malaysia, studies have implicated the primarily exophagic *Anopheles leucosphyrus* group of mosquitoes as the main vector of *P. knowlesi* and have found relatively high biting rates in farm edges bordering forests and forest areas (*11**–**14*). Human-disturbed environments have been associated with changes in behavior of nonhuman primates and their increased contact with humans (*15*). Fragmentation of existing habitats can also increase the frequency of disease transmission by creating transition areas with increased spatial overlap among human, mosquito, and wildlife populations or by altering vector ecology (*16**,**17*). The effects of these changes at forest edges have been described for malaria and other vector-borne zoonotic diseases (*18**,**19*) but not for *P. knowlesi*. A previous mathematic modeling study highlighted the potential for increased transmission resulting from increased spatial overlap among people, macaques, and mosquitoes at forest edges (*20*).

Despite these apparent links between land use and *P. knowlesi* transmission, detailed environmental risk factors for *P. knowlesi* infections in humans are unknown. Although variability of *P. knowlesi* risk has been reported at a regional scale, patterns of *P. knowlesi* transmission have not been described at a subdistrict spatial scale (*2**,**21*). Furthermore, studies that formally evaluate associations between *P. knowlesi* and characteristics of environment and landscape are lacking.

After obtaining approval from the Medical Research and Ethics Committee of the Ministry of Health in Malaysia, we examined the changing incidence of *P. knowlesi* in Kudat and Kota Marudu districts in northwestern Sabah, Malaysia, on the island of Borneo, an area with relatively high *P. knowlesi* transmission (*22*). Our aim was to describe the spatial and temporal patterns of *P. knowlesi* incidence within these districts and to explore potential associations between village-level incidence and deforestation and other environmental factors. Clarifying these relationships is vital to predicting and responding to future disease outbreaks and understanding the underlying mechanisms of *P. knowlesi* emergence.

## Methods

### Study Site and Population

**Figure 1:**
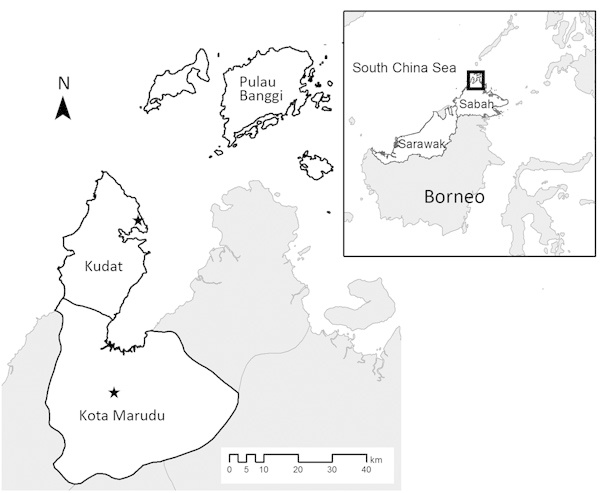
Location of Kudat and Kota Marudu districts in Sabah, Malaysia, where study of association of landscape and environmental factors and incidence of *Plasmodium knowlesi* transmission was conducted. Stars indicate location of the district hospitals. Inset shows location of these districts on the island of Borneo (box).

This study was conducted in the districts of Kudat and Kota Marudu in northwestern Sabah, Malaysia (07.38°–06.19°N, 116.62°–117.46°E), an area of 3,204 km^2^ with a population of ≈120,000 persons predominantly of Rungus and Dusun ethnicities (*23*) (Figure 1). The climate is tropical, with no dry season and increased rainfall during November–March; the area has both coastal and inland regions and elevations ranging from sea level to 1,000 m above sea level. Substantial environmental change is ongoing in the region because of conversion of land for oil palm plantations and other agricultural activities (*24*). Both districts have central referral hospitals that serve defined catchment areas where patients have access to diagnosis and treatment free of charge. All clinics refer patients to the central district hospital, where hospitalization is mandatory for malaria patients until a negative blood smear for malaria parasites has been obtained.

### Geolocation of Patients and Villages

We conducted a retrospective review of malaria patients reported by Kudat and Kota Marudu district hospitals during 2008–2012. Data on diagnosis, admission date, demographics, and address of all malaria patients were obtained from hospital laboratory microscopy records. Villages and populations were identified from the 2010 Population and Housing Census in Malaysia (*23*), the most recent census conducted in this area, and the global positioning system coordinates of village centroids were recorded as part of a larger interdisciplinary study (MONKEYBAR ESEI project; http://malaria.lshtm.ac.uk/MONKEYBAR). Village populations were updated by using published population growth rates for Malaysia (*25*). All locations were confirmed by using imagery available through Google Earth (https://www.google.com/earth/) or other freely available satellite data. Patient addresses were matched to the census data or to the nearest reported village from the census data. Administrative boundaries were used to define the extent of urban areas, within which village data were combined.

### Calculating the Proportion of Malaria Patients with *P. knowlesi*

*P. knowlesi* is microscopically similar to *P. malariae* but can also be misdiagnosed as *P. falciparum* or other human malaria species. In hospital microscopy records, the species of malaria is recorded, as determined by morphology, but no separate listing for *P. knowlesi* exists. Consequently, uncertainty in diagnosis resulted in some infections being recorded as *P. malariae* or *P. malariae/knowlesi*. To estimate the true proportion of *P. knowlesi*, we calculated the sensitivity and specificity of microscopy diagnosis of *P. malariae* for a subset of 539 malaria patients for whom both microscopy and molecularly confirmed results were available, including all patients from Kudat and Kota Marudu hospitals who were referred to a tertiary care hospital during this period and all patients recruited at these hospitals during 2013 and 2014 (*22**,**26*). The proportion of malaria cases reported as *P. knowlesi* per village per year was adjusted for this sensitivity and specificity by using a Bayesian estimation of true incidence from apparent incidence obtained by testing individual samples (*27*). This estimation is calculated as follows:

Models were fitted in R (http://www.R-project.org) through the prevalence and rjags packages interfacing with JAGS version 3.4.0 (http://mcmc-jags.sourceforge.net/) by using 2 chains containing 1,000 burn-in samples and 5,000 retained samples. Sensitivity and specificity parameters were determined by using β-PERT distributions of the minimum, maximum, and most likely values.

### Environmental Data

Topography and land use data were extracted from various datasets derived from satellite-based remote-sensing data. These data were evaluated for buffer areas (i.e., areas within a certain radius of a village center) with a radius of 1, 2 and 5 km from the center of each village; these distances were chosen to explore a range of spatial scales at which environment may be relevant on the basis of the typical distribution of households, farming land, and local human and animal movements. Elevation data with a spatial resolution of 30 m were obtained from the ASTER Digital Global Elevation Model (*28*). The average annual normalized difference vegetation index (NDVI), which quantifies the greenness of vegetation, was calculated from Moderate Resolution Imaging Spectroradiometer 16-day composites at 250-m resolution (*29*). The NDVI is influenced by climatic factors (e.g., rainfall and temperature) and has been used extensively to predict malaria incidence and develop early warning systems in other contexts (*30**,**31*).

Tree cover data, derived from classified Landsat imagery at 30-m resolution, were obtained from Hansen et al. (*32*). Annual forest cover maps for the study districts were produced; forest was defined as >50% tree crown cover density. Although this land classification represents forested areas, it cannot distinguish types of forest or agroforestry such as rubber or oil palm. The proportion of forest coverage, proportion of forest loss during the year for which incidence was estimated, and proportion of cumulative forest loss for the previous 5 years (i.e., total forest loss for 2006–2010 was evaluated by using 2010 incidence) were calculated for each buffer radius for each village and time point. We used the Landscape Ecology Statistics plugin for Quantum GIS (*33*) to evaluate the effect of forest configuration as the number of forest patches per radius, a standard metric representing landscape fragmentation. Distributions of these variables were examined, and quartiles were used to categorize variables.

Because this analysis relied on passive reporting of malaria, we included travel time to the nearest clinic where patients would seek treatment for a febrile illness as a measure of access to care. Travel time to the clinic from each village was estimated by using travel times reported in community interviews and by patients recruited as part of a population-based case–control study (*26*).

### Statistical Analysis

Annual *P. knowlesi* incidence for each village was mapped and smoothed incidence maps produced to visualize the data by using a kernel density estimation method, a standard method for interpolating point location data. Because the data were skewed relative to Poisson distribution, potential associations between environmental factors and reported *P. knowlesi* patients at the village level were assessed by using general linearized mixed models with a negative binomial distribution and an offset for population in R (*34*). To account for correlation between repeat measurements for the same village, we included the village variable as a random effect. Bivariable analysis was conducted for each covariate; variables for which p<0.2 were included in multivariable models. We used likelihood ratio tests to assess the significance of single variables and the Akaike Information Criterion (http://www.modelselection.org/aic/) for the final model selection. For correlated variables (e.g., mean elevation at different buffer radiuses), a single variable was selected for inclusion on the basis of marginal Akaike Information Criterion values. Potential bias from residual spatial autocorrelation in the model was explored for island and mainland areas through Moran’s I. On the basis of this statistic, the negative binomial model was fit with a spatial correlation component estimated by using a distance-based Matern correlation function.

## Results

### Malaria in Kudat and Kota Marudu Districts from 2008–2012

A total of 405 villages were mapped in Kudat and Kota Marudu districts; median population per village was 168 persons (interquartile range 80–313), and median number of households was 44 (interquartile range 20–78). A total of 2,006 malaria patients were reported during 2008–2012: 833 cases in Kota Marudu district, 1,014 in Kudat district, and 159 from outside these districts. Standard reporting forms did not include age-specific data but classified patients as adults (65.7%, 1,318/2,006) or children (32.8%, 657/2,006), with a small number of records (1.5%, 31/2006) missing this information. Most malaria patients were male (66%, 1,330/2,006). 

Most villages (60%, 245/405) reported at least 1 malaria patient during this period. The number of malaria patients reported varied annually, with marked seasonal variations in numbers of patients and amount of rainfall. Almost half (47%, 878/1,847) of reported malaria patients had suspected *P. knowlesi* or *P. malariae* infections diagnosed by microscopy. *P. falciparum* and *P. vivax* malaria were diagnosed in another 27% (512/1,847) and 25% (457/1,847) of patients, respectively.

Of 346 samples collected from patients for whom *P. knowlesi or P. malariae* was diagnosed by microscopy (including mixed infections) and sent for molecular confirmation, 90% (313/346) were confirmed as *P. knowlesi* by PCR. Sensitivity and specificity of microscopy diagnosis for *P. knowlesi* were 95% (95% CI 92%–97%) and 84% (95% CI 79%–89%), respectively (Table 1). Although studies have reported frequent misdiagnosis (*1**–**3**,**5*), few samples (3%, 16/539) were incorrectly identified as other species by microscopy. Because PCR results were available only for patients with confirmed malaria, we could not estimate the probability of detecting submicroscopic infections.

**Table 1:** Comparison of results of PCR and microscopy testing for *Plasmodium knowlesi*, Sabah, Malaysia, 2008–2012*

Microscopy results	No. samples		
	*P. knowlesi* PCR +	*P. knowlesi* PCR –	Total
*P. malariae* +	313	33	346
*P. malariae* –	16	177	193
Total	329	210	539

*+, positive; –, negative.

**Figure 2:**
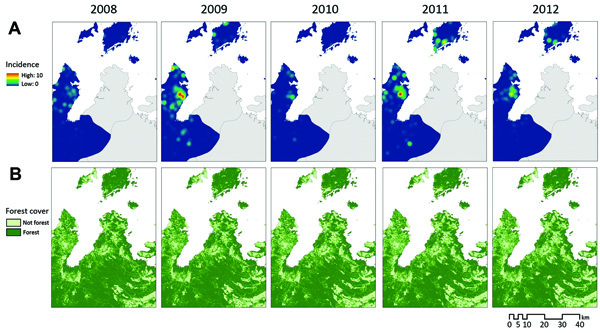
Comparison of estimated incidence (per 1,000 population) of *Plasmodium knowlesi* infection (A) and forest cover (B) and, Sabah, Malaysia, 2008–2012.

By using these values derived from collected samples, the true number of *P. knowlesi* patients was estimated as 739 (95% CI 664–794) for Kudat and Kota Marudu districts during 2008–2012. The range of estimated annual parasite incidence (API, expressed as cases/1,000 person/y) for *P. knowlesi* malaria calculated for each village was 0–102; overall mean API was 1.84. Of the 245 villages reporting malaria patients, 24% (59/245) had an estimated mean API for *P. knowlesi* of <l; the highest proportion, 44% (108/245), each had a mean API of 1–5; another 11% (26/245) had a mean API of 5–10; and 6% (15/245) had a mean API of 10–20. Two villages had a mean API of >20 (Figure 2, panel A).

### Association with Environmental Variables

On the basis of estimates from remote sensing data (*32*), forest cover declined by 4.8% in Kudat and Kota Marudu during 2008–2012 (Figure 2, panel B). Although this loss was often highly localized, large tracts of forest were cleared in the interior of Pulau Banggi. Overall, substantial environmental change was observed in the districts; 39% (157/405) of villages lost >10% of forest cover within a 1-km radius during this 5-year period; 44% (179/405) lost >10% within a 2-km radius; and 51% (206/405) lost >10% within a 5-km radius.

Lower elevations, >65% of forest cover (within a 2-km radius), and higher historical forest loss were associated with greater incidence of *P. knowlesi* infections. Travel times to clinics, average annual NDVI, and number of forest patches were not significant at any radius in the bivariate analysis.

### Spatial Patterns

Maps of interpolated *P. knowlesi* incidence show distinctive spatial patterns that vary from year to year, with some areas of high incidence persisting over time (Figure 2, panel A). To assess the degree of residual spatial correlation after fitting the negative binomial regression, the residuals of the best-fitting model were mapped and Moran’s I was calculated (mainland Borneo, Moran’s I 0.07, p<0.0001; islands off Borneo’s coast, Moran’s I 0.07, p = 0.07). Because Moran’s I was significant, the final model was adjusted for spatial autocorrelation (Tables 2, 3).

**Table 2:** Land use characteristics and bivariable statistics in *Plasmodium knowlesi* study, Sabah, Malaysia, 2008–2012*

Variable	Bivariable analysis	
	IRR (95% CI)	p value
% Forest remaining		
≤1 km radius		
<65	Reference	0.59
≥65	1.08 (0.82–1.43)	
≤2 km radius		
<65	Reference	0.06
≥65	1.30 (0.99–1.71)	
≤5 km radius		
<65	Reference	0.77
≥65	1.05 (0.78–1.40)	
% Forest lost in current year†		
≤1 km radius		
<1	Reference	0.11
1–2	0.94 (0.74–1.19)	
>2	1.22 (0.99–1.51)	
≤2 km radius		
<1	Reference	0.001
1–2		
>2	1.36 (1.09–1.71)	
≤5 km radius		
<1	Reference	0.01
1–2	0.97 (0.75–1.27)	
>2	1.57 (1.19–2.06)	
% Forest lost in past 5 years‡		
≤1 km radius		
<8	Reference	0.60
8–14	1.01 (0.76–1.33)	
>14	1.14 (0.85–1.53)	
≤2 km radius		
<8	Reference	<0.001
8–14	1.66 (1.25–2.20)	
>14	2.03 (1.46–2.80)	
≤5 km radius		
<8	Reference	0.32
8–14	0.93 (0.69–1.25)	
>14	1.13 (0.81–1.58)	
Mean elevation per 10 m above sea level		
≤1 km radius	0.99 (0.97–1.00)	0.12
≤2 km radius	0.98 (0.97–1.00)	0.06
≤5 km radius	0.97 (0.96–0.99)	0.01
Travel time to clinic, min	1.00 (0.99–1.00)	0.001

*Radius is calculated as distance from village center. IRR, incident rate ratio. †Current year is the year for which incidence was calculated. ‡Five years before the specific year of reported incidence.

**Table 3:** Multivariable negative binomial regression of *P. knowlesi* incidence (including population offset) in *Plasmodium knowlesi* study, Sabah, Malaysia, 2008–2012*

Variable	IRR (95% CI)	p value
% Forest remaining, ≤2 km radius		
<65	Reference	0.0004
≥65	1.51 (1.42–1.99)	
% Forest lost in the past 5 y, ≤2 km radius		
<8	Reference	<0.0001
8–14	1.68 (1.27–2.22)	
>14	2.22 (1.53–2.93)	
Mean elevation per 10 m above sea level, ≤5 km radius	0.98 (0.96–0.99)	0.001

*Population was used as an offset to correct the number of infections reported for an estimate of the population size. Radius is calculated as distance from village center. IRR, incident rate ratio.

## Discussion

This study aimed to describe spatial and temporal patterns of *P. knowlesi* infection in northern Sabah, Malaysia, and to evaluate potential associations between village-level *P. knowlesi* incidence and key environmental factors. Although land use changes can affect emergence of infectious diseases, such associations have not been previously evaluated for *P. knowlesi.* We found links among deforestation, environmental characteristics, and reported incidence of *P. knowlesi* malaria in this region.

Village-level malaria data show marked spatial and temporal heterogeneity in *P. knowlesi* incidence in Kudat and Kota Marudu districts. After adjustment for the sensitivity and specificity of microscopy, *P. knowlesi* was the most common cause of human malaria, a finding consistent with other studies for this geographic area (*4**,**5**,**22*). Quantification of annual forest loss indicates that substantial environmental changes occurred during 2008–2012, with many villages losing substantial proportions of surrounding forest cover.

The proportion of forest surrounding a village was associated with incidence of *P. knowlesi* infections in the final model (Table 3); this association potentially reflects the role of forest environments as habitats of macaques and mosquito vectors. Studies report higher vectorial capacity and sporozoite rates for *P. knowlesi* in forest environments than in agricultural and settled lands (*12**,**35*). Long-tailed macaques have also been reported in various environments, including degraded secondary forest areas (*36*). The association of increased *P. knowlesi* incidence with both forest and forest loss likely confirms findings that transmission is occurring in forested areas undergoing substantial change, as with previously described frontier malaria (*37*).

Higher incidence of *P. knowlesi* was associated with higher proportions of forest loss surrounding villages during the 5-year period before year of reported incidence. This association could result from changes in macaque or mosquito habitats and from increased levels of human activity. Increased density of long-tailed macaques has been reported as a response to deforestation; loss of previous habitats can result in crowding within forest patches, with potential implications for disease transmission (*36*). Land use changes have also been shown to affect abundance and community composition of potential vectors (*17**,**38*). Deforestation and associated agricultural development are also associated with changes in human risk because of altered distribution and behavior of humans; these changes result from more employment opportunities and shifts in human movement patterns because of forest clearing and agricultural activities (*19**,**37*). Although clearing of forests may initially deplete vector populations and thereby reduce malaria transmission, this reduction may be followed by colonization of cleared areas by more efficient vector species and subsequent increases in transmission (*9*). Historical forest loss was more significantly associated with *P. knowlesi* incidence than forest loss occurring during the same year of reported incidence (Table 2), suggesting that increased transmission is related to long-term changes in vector, host, or human populations involved. Additional longitudinal studies are required to investigate this hypothesis.

The effect of habitat may also be reflected in associations with elevation. Elevation was negatively correlated with *P. knowlesi* incidence, although the range of elevations within the study site was limited; most villages were at elevations <100 m. Both macaques and vectors are more frequently described in low elevation areas but have been reported at higher elevations (*36**,**39*).

Neither forest configuration nor vegetation indices appeared to be strongly associated with *P. knowlesi* incidence. Because NDVI is a measure of vegetation greenness, this measure may not differentiate between forest and other types of agriculture (e.g., oil palm) because NDVI tends to become saturated in tropical environments. Studies of other zoonotic diseases have found that the fragmentation and configuration of other types of land cover, in addition to forest, influence disease transmission (*18**,**19*). Other fragmentation metrics could be included to evaluate the effects of forest patch size and shape on *P. knowlesi* transmission.

The buffer sizes that we evaluated represent a range of potential scales at which variables (e.g., mean elevation) within different radiuses may affect *P. knowlesi* transmission. Human infections result from many factors interacting across different spatial scales, and the strength of association of these factors likely varies by distance. Our study covers an extensive area in which behaviors, distribution of villages, and ecology vary. Villages are typically within small spatial areas, but farming practices range from small-scale swidden farming to large-scale plantations and affect the scale of human interactions with the environment. Future analyses would benefit from including more detailed spatial data on household locations and human movement patterns.

The main limitation of this study is the reliance on records of malaria patients who sought care at hospitals; these patients may represent only a portion of malaria patients in the community. Although malaria is a notifiable disease and distance to a hospital was included as a measure of access to care, asymptomatic malaria cases and symptomatic cases that resolved without treatment are unaccounted for. Previous studies of other malaria species in similar transmission settings have described a large proportion of asymptomatic carriage within communities; however, asymptomatic carriage has not been evaluated for *P. knowlesi* (*40*). A cross-sectional survey of 2,019 persons in central Vietnam identified 3 persons whose samples were positive for *P. knowlesi*, yet all 3 were asymptomatic at the time of the survey and for the subsequent 6 months, showing that asymptomatic carriage of *P. knowlesi* can occur (*40*).

This study was also limited by the environmental data used. Data on forest cover and forest loss were aggregated by year, and finer-scale temporal associations between land cover (i.e., forest and other land types) and incidence could not be explored. This dataset was limited by defining forest as canopy cover, a definition that does not enable differentiation between types of forest or crops or between patches of different types of land. In addition, analysis was limited by the spatial resolution of satellite-based remote-sensing data, the use of a centroid point to represent village location, and the use of circular buffers rather than buffers of actual village shape. Exploratory spatial analysis suggested spatial heterogeneity in village-level data, and these spatial effects were included in the model. Additional work with more spatially specific outcomes and environmental data are needed to investigate these spatial patterns in more detail.

Despite inherent limitations in the outcome and covariate data used in this study, results strongly suggest a link between environmental change and reported incidence of emerging *P. knowlesi* in northern Sabah. Spatial analysis of environmental factors affecting disease emergence can be used to target surveillance and public health activities to areas expected to have increasing disease risk. Although additional population-based studies are needed to define environmental risk factors, this study indicates that deforestation is associated with human cases of *P. knowlesi* within northwestern Sabah, Malaysia.
